# Elamipretide in pediatric Barth syndrome: from heart failure to school return

**DOI:** 10.1186/s13023-026-04487-z

**Published:** 2026-07-07

**Authors:** Pascal Amedro, Mathieu Andrianoely, Pauline Gohier, Justine Leroux, Nathalie Aladjidi, Stephanie Lagorce, Melissa Daccord, Marion Audie, Astrid Botte, Karine Nubret, Pascale Richard, Sandrine Roche, Delphine Lamireau, Candice Tetard, Aurelien Trimouille, Arielle Desir, Samir Mesli, Jean Donadieu, Julien Gotchac

**Affiliations:** 1 Department of Pediatric and Congenital Cardiology, CRMR-M3C, National Reference Center for Complex Congenital Heart Disease, Bordeaux University Hospital, IHU Liryc, INSERM U1045, University of Bordeaux, Bordeaux, France; 2 Heart and Lung Center, Heart Research Institute, Children’s National Hospital, The George Washington University School of Medicine & Health Sciences, 111 Michigan Ave NW, Washington, DC 20010-2916 USA; 3 Just Coaching, Paris, France; 4 Clinical Pharmacy Department, Haut-Leveque Hospital, Bordeaux University Hospital, Bordeaux, France; 5 Pediatric Hemato-Immunology, CIC1401, INSERM CICP, National Reference Center for Autoimmune Cytopenia in Children (CEREVANCE), Bordeaux University Hospital, Bordeaux, France; 6 Le Hillot Cardiac Rehabilitation Center, Pessac, France; 7 Pediatric Home Hospitalization Unit (HAD), Le Bouscat Suburban Hospital, Le Bouscat, France; 8 Department of Cardiology and Cardio-Vascular Surgery, Pediatric Heart Transplant Unit, Bordeaux University Hospital, Bordeaux, France; 9 Functional Unit of Molecular and Cellular Cardiogenetic and Myogenetic, Metabolic Biochemistry, Assistance Publique–Hôpitaux de Paris (AP-HP), Pitié Salpêtrière Hospital, INSERM U1166, Sorbonne University and ICAN, IHU of Cardiometabolism, Paris, France; 10 Department of Pediatric Gastroenterology and Nutrition, Bordeaux University Hospital, Bordeaux, France; 11 Department of Medical Genetics, Bordeaux University Hospital, Bordeaux, France; 12 INSERM U1211 – Rare Diseases: Genetics and Metabolism (MRGM), University of Bordeaux, Bordeaux, France; 13 Laboratory of Biochemistry for Inherited Metabolic Diseases, Bordeaux University Hospital, Bordeaux, France; 14 Department of Pediatric Hemato-Immuno-Oncology, Neutropenia Registry, Chronic Neutropenia Reference Center, Armand-Trousseau Hospital, Assistance Publique–Hôpitaux de Paris (AP-HP), Paris, France

**Keywords:** Barth syndrome, Elamipretide, Cardiac function, Exercise tolerance, Nutrition, Health-related quality of life, Cardiac rehabilitation

## Abstract

**Background:**

Barth syndrome (BTHS) is a rare X-linked mitochondrial disorder characterized by cardiomyopathy, neutropenia, and skeletal myopathy. Elamipretide is a mitochondria-targeting peptide that stabilizes cardiolipin and improves mitochondrial function. While use of this treatment in infants with BTHS has been reported in the United States, no cases have been described outside the US or in older children.

**Case presentation:**

We report the first case of a preschool-aged child with genetically confirmed BTHS and advanced heart failure, who was treated with elamipretide under a compassionate use program approved by the French Drug Administration (ANSM). At treatment initiation, this 5-year-old patient presented with severe left ventricular dysfunction (LVEF = 20%) and moderate-to-severe mitral regurgitation. He was dependent on gastrostomy feeding and had been listed for heart transplantation. Subcutaneous elamipretide (10 mg/day) was initiated alongside maximal conventional heart failure therapy. Over six months, LVEF improved to 50%, mitral regurgitation decreased to mild, Ross class improved from IV to I, and substantial gains were observed in functional capacity, nutritional status, and health-related quality of life (HRQoL). The patient returned to full-time school and remained temporarily suspended from the transplant list. Treatment was generally well tolerated. One transient, non-serious episode of abdominal pain with moderate pancreatic enzyme elevation occurred, which resolved without treatment discontinuation.

**Conclusions:**

This case suggests that elamipretide may improve cardiac function, exercise tolerance, nutrition, and HRQoL in preschool-aged pediatric BTHS patients, potentially delaying the need for transplantation. Broader pediatric evaluation is warranted. Further strategies, such as youth-tailored hybrid cardiac rehabilitation programs, could complement pharmacological therapy and further optimize outcomes in this population.

## Background

Barth syndrome [MIM:302060] is a rare X-linked recessive disorder caused by pathogenic variants in the *TAFAZZIN* gene, which encodes an acyltransferase essential for remodeling mature cardiolipin in the inner mitochondrial membrane [1]. Abnormal cardiolipin composition leads to mitochondrial dysfunction, resulting in a characteristic phenotype that may include dilated or hypertrophic cardiomyopathy, neutropenia, skeletal myopathy, growth delay, and exercise intolerance [1–3]. The estimated prevalence is approximately 1 in 1 million live male births [4]. Cardiomyopathy typically presents in infancy and carries a high risk of death or heart transplantation in early childhood [3].

No curative therapy is currently available for Barth syndrome in children. Management remains supportive, combining guideline-directed medical therapy for heart failure, prophylaxis for neutropenia, and close monitoring for arrhythmias.

Elamipretide is a mitochondria-targeting tetrapeptide that binds cardiolipin, stabilizes the inner mitochondrial membrane, improves electron transport chain efficiency, enhances adenosine triphosphate (ATP) synthesis, and reduces reactive oxygen species production [5–7]. In the phase 2/3 TAZPOWER trial and its open-label extension, elamipretide improved functional capacity and left ventricular systolic function in adolescents and young adults with Barth syndrome [8, 9]. Expanded-access use has been reported in two critically ill pediatric patients in the United States—a newborn [10] and an 11-month-old infant [11]—both of whom showed marked recovery of ventricular function after treatment initiation.

To our knowledge, no pediatric case of elamipretide therapy has been reported in older children or outside the United States. We describe the first European case of elamipretide treatment in a child with genetically confirmed Barth syndrome, initiated at 5 years of age, with comprehensive follow-up including cardiac imaging, functional capacity testing, and health-related quality of life (HRQoL) assessment.

## Case presentation

A male child with genetically confirmed Barth syndrome was followed at our tertiary pediatric cardiology center from infancy for dilated cardiomyopathy with severely reduced left ventricular ejection fraction (LVEF) and progressive exercise intolerance. He was the first child of non-consanguineous parents. He had been evaluated at the age of 2 years in pediatric hematology for asymptomatic transient neutropenia (0.4–0.7 G/L).

His younger brother had died at 14 months of age from fulminant dilated cardiomyopathy with cardiogenic shock refractory to maximal therapy, culminating in cardiac arrest in a septic context. Despite resuscitation and mechanical circulatory support, he died of multiorgan failure. Metabolic investigations revealed significant 3-methylglutaconic aciduria. In light of this familial history, Barth syndrome was suspected in the index patient, prompting cardiologic and genetic investigations.

The present patient underwent cardiology assessment at 3 years and 8 months as part of family screening. Echocardiography revealed an LVEF of 50% with mild ventricular dilatation. The combination of cardiac involvement and neutropenia prompted targeted genetic testing, confirming Barth syndrome. Molecular analysis by NGS panel of 72 genes involved in cardiomyopathies identified a hemizygous variant in the TAFAZZIN gene (NM_000116.5: c.278del; p.(Met93Serfs*46)) located in exon 3, causing a frameshift with a premature stop codon. This variant had not been reported in public databases (including GnomAD v4.1.0), and in silico predictions supported a deleterious effect. According to American College of Medical Genetics and Genomics (ACMG) criteria, it was classified as Likely Pathogenic (Class 4 PVS1, PM2). An analysis of the *TAFAZZIN* gene was performed on the asymptomatic patient’s mother, revealing the same mutation in a heterozygous state. Both parents had normal echocardiography, and no other affected relatives were identified.

The patient developed progressive deterioration of cardiac function. At 4 years, cardiac MRI revealed an LVEF of 35% with moderate dilation, prompting initiation of standard heart failure therapy. At 5 years, his condition had progressed to Ross class IV, with nasogastric feeding due to loss of oral intake. Echocardiography showed LVEF of 20% and moderate-to-severe mitral regurgitation. Despite optimal therapy, intermittent intravenous furosemide was required during decompensation. Feeding difficulties and malnutrition led to gastrostomy placement. Neutrophil counts remained normal under antibiotic prophylaxis. He was subsequently listed for heart transplantation (Table 1).

**Table 1:** Patient follow-up data before and after elamipretide initiation

	Diagnosis	Decompensated heart failure	Elamipretide initiation	1-month treatment	3-month treatment	6-month treatment
**Clinical data**						
Age (years and months)	4 years 1 month	4 years 10 months	5 years 5 months	5 years 6 months	5 years 8 months	5 years 11 months
Weight, Kg (Z-score)	12 (-3.0)	14 (-2.2)	14.9 (-2.2)	15 (-2.3)	15.9 (-1.9)	18.1 (-0.9)
Height, cm (Z-score)	92 (-2.6)	94 (-3.0)	97 (-3.0)	97 (-3.1)	97 (-3.3)	99 (-3.1)
Body mass index, kg/m^2^ (Z-score)	14.2 (-1.5)	15.8 (+ 0.3)	15.8 (+ 0.4)	15.9 (+ 0.4)	16.9 (+ 1.0)	18.5 (+ 1.7)
Waist circumference (cm)	-	-	52	-	-	55
Blood pressure: systolic/diastolic (mmHg)	79/47	88/51	78/40	75/44	82/42	90/54
ROSS functional class	I	IV	III	II	I	I
**Patient care**						
Feeding mode	Normal (difficulties in oral intake)	Cessation of oral intake, nasogastric tube	Full feeding from gastrostomy	2/3 gastrostomy, 1/3 oral intake	1/3 gastrostomy, 2/3 oral intake	Full oral intake (gastrostomy button remained in place for safety)
Follow-up modality	Outpatient pediatric cardiology clinic	Inpatient pediatric cardiology clinic	Inpatient pediatric cardiology clinic	Home hospitalization + weekly outpatient pediatric cardiology clinic	Home hospitalization + bi-monthly outpatient pediatric cardiology clinic	Home hospitalization + monthly outpatient pediatric cardiology clinic
Medication	None	Furosemide (intravenous), angiotensin-converting enzyme inhibitors, beta-blockers, mineralocorticoid receptor antagonists, trimethoprim-sulfamethoxazole	Elamipretide 10 mg/day (subcutaneous), furosemide (oral), angiotensin-converting enzyme inhibitors, beta-blockers, mineralocorticoid receptor antagonists, anticoagulants (warfarin), trimethoprim-sulfamethoxazole	Elamipretide 10 mg/day (subcutaneous), furosemide (oral), angiotensin-converting enzyme inhibitors, beta-blockers, mineralocorticoid receptor antagonists, anticoagulants (warfarin), trimethoprim-sulfamethoxazole	Elamipretide 10 mg/day (subcutaneous), furosemide (oral), angiotensin-converting enzyme inhibitors, beta-blockers, mineralocorticoid receptor antagonists, aspirin, trimethoprim-sulfamethoxazole	Elamipretide 10 mg/day (subcutaneous), furosemide (oral), angiotensin-converting enzyme inhibitors, beta-blockers, aspirin, trimethoprim-sulfamethoxazole
Heart transplant list	No	Pending	Yes	Yes	Temporary suspension	Temporary suspension
School	Full-time	Out of school	Out of school	Part-time	Full-time	Full-time
**Electrocardiogram**						
Heart rate at rest (bpm)	111	126	91	90	100	88
PR interval (msec)	138	177	162	150	150	140
QRS duration (msec)	66	90	105	80	80	80
QRS axis (°)	9	-2	-6	0	-7	0
QTc interval	390	440	450	410	430	410
24-hour Holter monitoring	-	No arrythmia	No arrythmia	-	-	-
**Transthoracic echocardiography**						
LVEF (%)	40	20	25	35	40	50
End diastolic LV, mm (Z-score)	35 (+ 2.3)	49 (+ 6.9)	43 (+ 4.4)	43 (+ 4.4)	42 (+ 3.8)	39 (+ 2)
Mitral regurgitation	None	Moderate-to-severe	Moderate-to-severe	Mild	Mild	Mild
Systolic pulmonary artery pressure (mmHg)	22	37	30	No tricuspid valve regurgitation	No tricuspid valve regurgitation	No tricuspid valve regurgitation
**Biological data**						
NT-proBNP (pg/mL)	1114	29 444	2466	-	1152	419
Neutrophil count (× 10⁹/L)	3.18	0.42	3.35	-	3.62	4.65

Legend: Clinical, biological, electrocardiographic, and echocardiographic parameters are reported from the time of diagnosis, during decompensated heart failure, and after initiation of subcutaneous elamipretide therapy

LVEF, left ventricular ejection fraction; LV, left ventricle; NT-proBNP, N-terminal pro-B-type natriuretic peptide

Given the severity and lack of alternatives, elamipretide was initiated under the Expanded Access Program, with approval from the French Drug Administration (“Agence Nationale de Sécurité du Médicament et des produits de santé”, ANSM) as compassionate use within the European Rare Disease Plan, following parental consent.

## Intervention and follow-up

At baseline and after 6 months of treatment, clinical status (Ross functional class, feeding mode, anthropometric data), electrocardiography, echocardiography, physical fitness, and patient-reported outcomes were assessed. Physical fitness was evaluated in terms of functional capacity (six-minute walk test) [12], maximum isometric strength of the upper-limb (handgrip test) [13] and the lower-limb (standing long jump test) [14], static balance (one-leg stance test), speed/agility (4 × 10 m shuttle run test) [15, 16], and physical activity. A triaxial accelerometer (ActiGraph GT3X, ActiGraph LLC) was used to assess physical activity levels, offering objective data on moderate-to-vigorous physical activity (MVPA) [17]. Adherence was evaluated according to the latest World Health Organization (WHO) guidelines, which recommend at least 60 min per day of MVPA for children [18]. HRQoL was measured with the Pediatric Quality of Life Inventory (PedsQL) 4.0 Generic Core Scales, both self- and proxy-reported [19]. 

**Fig. 1:**
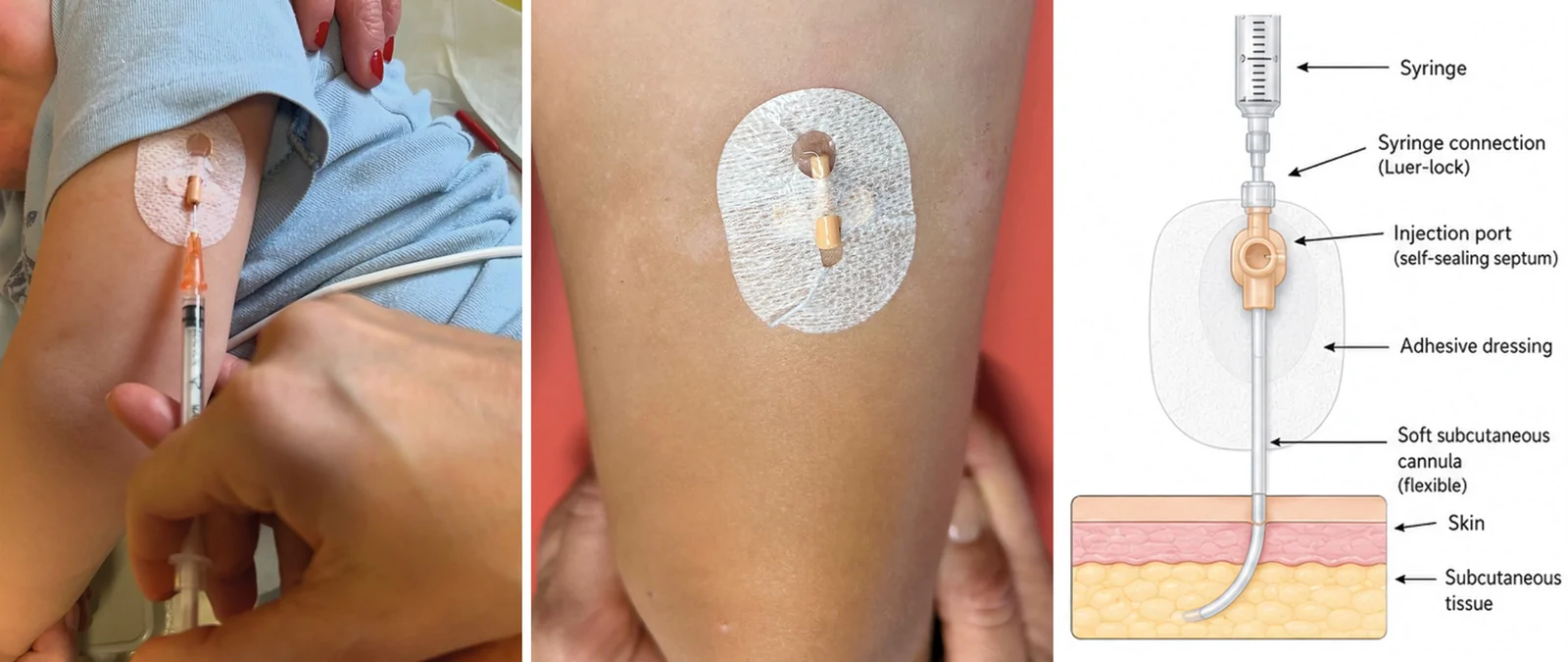
Subcutaneous administration of elamipretide. The Insuflon^®^ subcutaneous catheter was initially placed on the upper lateral aspect of the arm (left image), which was better accepted in the pediatric setting, and subsequently on the thigh (middle image) in accordance with manufacturer recommendations. The device was replaced weekly to minimize discomfort and avoid daily needle punctures, enabling safe home administration under pediatric home hospitalization (HAD) supervision. The schematic representation on the right illustrates the main components of the Insuflon^®^ device, including the injection port, adhesive dressing, and flexible subcutaneous cannula used for repeated subcutaneous administration

Elamipretide was administered subcutaneously at a fixed dose of 10 mg once daily (0.13 mL of an 80 mg/mL formulation), according to the manufacturer’s Expanded Access Program dosing recommendations for children weighing 10–20 kg, using an Insuflon^®^ subcutaneous catheter. During the first week, the device was placed in the upper arm for better tolerability in a young, active child. Thereafter, in accordance with manufacturer recommendations, it was placed in the thighs, alternating sides weekly, for the remainder of the treatment period (Fig. 1). Follow-up was coordinated with the local pediatric home hospitalization (“HAD”) team, which monitored the patient and participated in weekly cardiology staff videoconferences. The first injections were performed under inhaled nitrous oxide/oxygen mixture (MEOPA) without additional analgesia. Application of an ice pack at the injection site reduced local burning sensations. Thereafter, injections were consistently well tolerated without adverse events.

At 1 month of therapy, LVEF improved from 20% to 35%, mitral regurgitation decreased from moderate-to-severe to mild, Ross class improved from IV to II, partial oral intake resumed, and part-time school attendance was possible. At 3 months of therapy, LVEF reached 40%, mitral regurgitation improved to mild, Ross class improved to I, full-time school resumed, weight gain was adequate, and gastrostomy weaning began; transplant listing was temporarily suspended. At that time, the patient presented with low-grade fever, abdominal pain, and a moderate transient increase in pancreatic enzymes (lipase 356 U/L). Symptoms regressed within 48 h, abdominal ultrasound was normal, and treatment was maintained without interruption. Serology confirmed *Mycoplasma pneumoniae* primary infection, supporting an intercurrent infectious origin rather than drug-induced pancreatitis.

At 6 months, Ross class was I, oral feeding was nearly complete, LVEF was 50%, left ventricle was moderately dilated (+ 2 Z-score), and mitral regurgitation became mild (Table 1). Substantial nutritional recovery was observed, with weight increasing from 14.9 kg to 18.1 kg and corresponding improvement in age-adjusted anthropometric Z-scores. Functional capacity improved across all tested domains, with substantial gains in walking distance, muscular strength, balance, and agility. Accelerometer-derived daily moderate-to-vigorous physical activity (MVPA) remained below the WHO guideline recommendation of ≥ 60 min/day at both baseline and 6-month follow-up. HRQoL scores improved markedly in both self- and proxy-reports, particularly in physical functioning and psychosocial domains (Table 2). No other adverse events were reported during follow-up.

**Table 2:** Physical fitness, accelerometer-derived physical activity and health-related quality of life before and after 6 months of elamipretide therapy

		Elamipretide initiation	6-months follow-up	Change (absolute value)	Change (%)
**Physical fitness**					
Functional capacity	Six-minute walk test (m, %-predict)	90 (18.5)	310 (59.8)	+ 220 (+ 41.3)	+ 244.4%
Muscular strength	Upper limb muscular strength (right hand) – Handgrip test (kg)	2.9	3.2	+ 0.3	+ 10.3%
	Upper limb muscular strength (left hand) – Handgrip test (kg)	2.5	3.3	+ 0.8	+ 32.0%
	Lower limb muscular strength – Standing long jump test (cm)	35.3	45.6	+ 10.3	+ 29.2%
Static balance	Static balance on right foot – One-leg stance test (seconds)	3	3.5	+ 0.5	+ 16.7%
	Static balance on left foot – One-leg stance test (seconds)	2.2	3.3	+ 1.1	+ 50.0%
Speed/Agility	4 × 10 m shuttle run test (seconds)	29.7	24.6	−5.1	+ 17.2%
**Accelerometry-derived physical activity**					
Physical activity level	MVPA (minutes/day)	19	16	-3	-15.80%
	Meeting WHO guidelines (Yes/No)	No	No	-	-
**Health-related quality of life**					
Self-report (PedsQL)	Total score	58.7	67.4	+ 8.7	+ 14.8%
	Physical functioning	62.5	87.5	+ 25.0	+ 40.0%
	Psychosocial health summary score	56.7	56.7	0	0.00%
	Emotional functioning	60	40	−20.0	−33.3%
	Social functioning	30	60	+ 30.0	+ 100.0%
	School functioning	70	70	0	0.00%
Proxy-report (PedsQL)	Total score	50	77.2	+ 27.2	+ 54.4%
	Physical functioning	21.9	78.1	+ 56.2	+ 256.6%
	Psychosocial health summary score	65	76.7	+ 11.7	+ 18.0%
	Emotional functioning	50	65	+ 15.0	+ 30.0%
	Social functioning	75	80	+ 5.0	+ 6.7%
	School functioning	70	85	+ 15.0	+ 21.4%

Legend: Functional capacity, muscle strength, static balance, speed/agility, and health-related quality of life scores are reported for the patient at treatment initiation and at 6-month follow-up

MVPA, moderate-to-vigorous physical activity; PedsQL, Pediatric Quality of Life Inventory; WHO, World Health Organization

## Discussion

This is the first pediatric case of elamipretide therapy reported outside the United States, and the first in a preschool-aged child with Barth syndrome. Previous US reports described a newborn and an 11-month-old infant treated in acute, life-threatening contexts [10, 11]. In contrast, our patient presented with advanced chronic cardiomyopathy and progressive functional decline despite maximal medical therapy.

Although clinically significant cardiomyopathy became apparent later than is typically reported in Barth syndrome, no clear genotype–phenotype correlation has been established for TAFAZZIN loss-of-function variants. Notably, the patient’s younger brother, who carried the same pathogenic variant, developed severe infantile cardiomyopathy leading to death at 14 months of age. Consistent with previous cohort studies, substantial phenotypic variability has been reported among patients carrying truncating TAFAZZIN variants, irrespective of variant position within the gene [3].

Elamipretide was provided through the manufacturer’s Expanded Access Program, with approval from the French Drug Administration (ANSM), and delivered via the pediatric home hospitalization (HAD) team using a subcutaneous regimen specifically adapted to pediatric needs and compatible with ambulatory care. This mode of administration, using an Insuflon^®^ subcutaneous catheter, avoided daily needle punctures and improved treatment acceptability for the child and caregivers. It has been used successfully for other therapies in children [20, 21], but, to our knowledge, has not been previously reported for elamipretide. While the manufacturer recommends abdominal or thigh injection sites, the upper arm was initially selected during the first week for better acceptability in a young child, before transitioning to the thigh, consistent with official guidance. This illustrates the importance of tailoring administration strategies to pediatric patients to optimize tolerability and adherence in long-term subcutaneous therapy for rare diseases. Treatment was well tolerated overall, with only one transient, non-serious episode of suspected pancreatitis, most likely related to an intercurrent *Mycoplasma pneumoniae* infection rather than elamipretide itself. Therapy was not interrupted, and overall safety remained favorable throughout the 6-month follow-up.

Follow-up was multidimensional, combining echocardiography with mitral regurgitation grading, functional capacity testing, accelerometer-derived physical activity assessment, patient-reported outcome measures (PROMs), and nutritional assessment. Improvements were consistent across domains: LVEF rose from 20% to 50%, mitral regurgitation improved from moderate-to-severe to mild, Ross functional class improved from IV to I, and the patient returned to full-time school while remaining on temporary transplant suspension. Nutritionally, he progressed from exclusive gastrostomy feeding to near-complete oral intake. Functional outcomes, including walking distance, muscular strength, balance, and agility, showed substantial gains, and both self- and proxy-reported HRQoL improved markedly, particularly in physical functioning and psychosocial domains. Interestingly, self-reported emotional functioning scores declined during follow-up, whereas proxy-reported emotional functioning improved. Given the young age of the patient and the discordance between self- and proxy-reported assessments, this isolated finding should be interpreted cautiously and may reflect normal variability in emotional self-perception rather than a true deterioration in emotional well-being.

In young adults with Barth syndrome, physical exercise has been shown to improve muscle strength [22, 23], cardiorespiratory fitness [24], and quality of life [22, 24]. No such studies exist in children, but youth-tailored hybrid programs such as QUALIREHAB, combining center-based and home-based cardiac rehabilitation, could represent a promising approach to further optimize cardiac and muscular function in pediatric patients [25].

## Conclusions

Although spontaneous improvement cannot be excluded, the magnitude and timing of changes strongly suggest a therapeutic benefit of elamipretide. This observation supports further pediatric evaluation of elamipretide not only in acute neonatal presentations but also in older children with Barth syndrome and highlights the value of innovative delivery methods to enhance adherence and quality of life in long-term subcutaneous therapy for rare pediatric disorders. Youth-tailored cardiac rehabilitation could further enhance outcomes in pediatric Barth syndrome.

## Data Availability

The data and materials underlying this article will be shared on reasonable request to the corresponding author. The datasets supporting the conclusions of this article are included within the article.

## Abbreviations

ATP: Adenosine Triphosphate
ACMG: American College of Medical Genetics and Genomics
BTHS: Barth Syndrome
FDA: Food and Drug Administration
HRQoL: Health-Related Quality of Life
LVEF: Left Ventricular Ejection Fraction
MRI: Magnetic Resonance Imaging
MVPA: Moderate-to-Vigorous Physical Activity
MEOPA: Nitrous Oxide/Oxygen Mixture
PROMs: Patient-Reported Outcome Measures
HAD: Pediatric Home Hospitalization
ANSM: The French Drug Administration
WHO: World Health Organization

## References

[1] Clarke SL, Bowron A, Gonzalez IL, Groves SJ, Newbury-Ecob R, Clayton N et al. Barth syndrome. Orphanet J Rare Dis. 2013;8. 10.1186/1750-1172-8-23 PMC3583704 23398819

[2] Taylor C, Rao ES, Pierre G, Chronopoulou E, Hornby B, Heyman A, et al. Clinical presentation and natural history of Barth Syndrome: An overview. J Inher Metab Disea. 2022;45:7–16. 10.1002/jimd.12422. 34355402

[3] Rigaud C, Lebre A-S, Touraine R, Beaupain B, Ottolenghi C, Chabli A, et al. Natural history of Barth syndrome: a national cohort study of 22 patients. Orphanet J Rare Dis. 2013;8:70. 10.1186/1750-1172-8-70. PMC3656783 23656970

[4] Miller PC, Ren M, Schlame M, Toth MJ, Phoon CKL. A Bayesian Analysis to Determine the Prevalence of Barth Syndrome in the Pediatric Population. J Pediatr. 2020;217:139–44. 10.1016/j.jpeds.2019.09.074. 31732128

[5] Anzmann AF, Sniezek OL, Pado A, Busa V, Vaz FM, Kreimer SD, et al. Diverse mitochondrial abnormalities in a new cellular model of TAFFAZZIN deficiency are remediated by cardiolipin-interacting small molecules. J Biol Chem. 2021;297:101005. 10.1016/j.jbc.2021.101005. PMC8384898 34314685

[6] Sabbah HN, Gupta RC, Kohli S, Wang M, Hachem S, Zhang K. Chronic therapy with elamipretide (mtp-131), a novel mitochondria-targeting peptide, improves left ventricular and mitochondrial function in dogs with advanced heart failure. Circ Heart Fail. 2016;9. 10.1161/CIRCHEARTFAILURE.115.002206. PMC4743543 26839394

[7] Sabbah HN. Barth syndrome cardiomyopathy: targeting the mitochondria with elamipretide. Heart Fail Rev. 2021;26:237–53. 10.1007/s10741-020-10031-3. PMC7895793 33001359

[8] Reid Thompson W, Hornby B, Manuel R, Bradley E, Laux J, Carr J, et al. A phase 2/3 randomized clinical trial followed by an open-label extension to evaluate the effectiveness of elamipretide in Barth syndrome, a genetic disorder of mitochondrial cardiolipin metabolism. Genet Sci. 2021;23:471–8. 10.1038/s41436-020-01006-8. PMC7935714 33077895

[9] Thompson WR, Manuel R, Abbruscato A, Carr J, Campbell J, Hornby B, et al. Long-term efficacy and safety of elamipretide in patients with Barth syndrome: 168-week open-label extension results of TAZPOWER. Genet Sci. 2024;26:101138. 10.1016/j.gim.2024.101138. 38602181

[10] Ortmann L, Velasco D, Cole J. L <>MonzoNuzziBrunoKadíc V G F editors. 2025 Expanded-access use of elamipretide in a newborn with Barth syndrome: a case report. Eur Heart J - Case Rep 9 ytaf030 10.1093/ehjcr/ytaf030. PMC11799937 39917770

[11] Goldstein AC, Pantano C, Redko M, MacMullen LE, Maeda K, O’Connor MJ. Expanded-access use of elamipretide in a critically ill patient with Barth syndrome. Genet Med Open. 2024;2:101859. 10.1016/j.gimo.2024.101859. PMC11613783 39669625

[12] Geiger R, Strasak A, Treml B, Gasser K, Kleinsasser A, Fischer V, et al. Six-minute walk test in children and adolescents. J Pediatr. 2007;150:395–9. 10.1016/j.jpeds.2006.12.052. 399.e1-2. 17382117

[13] Gąsior J, Pawłowski M, Jeleń P, Rameckers E, Williams C, Makuch R, et al. Test–Retest Reliability of Handgrip Strength Measurement in Children and Preadolescents. IJERPH. 2020;17:8026. 10.3390/ijerph17218026. PMC7663254 33142693

[14] Castro-Piñero J, Ortega FB, Artero EG, Girela-Rejón MJ, Mora J, Sjöström M, et al. Assessing Muscular Strength in Youth: Usefulness of Standing Long Jump as a General Index of Muscular Fitness. J Strength Conditioning Res. 2010;24:1810–7. 10.1519/JSC.0b013e3181ddb03d. 20555277

[15] Ortega FB, Cadenas-Sánchez C, Sánchez-Delgado G, Mora-González J, Martínez-Téllez B, Artero EG, et al. Systematic Review and Proposal of a Field-Based Physical Fitness-Test Battery in Preschool Children: The PREFIT Battery. Sports Med. 2015;45:533–55. 10.1007/s40279-014-0281-8. 25370201

[16] Cadenas-Sanchez C, Martinez-Tellez B, Sanchez-Delgado G, Mora-Gonzalez J, Castro-Piñero J, Löf M, et al. Assessing physical fitness in preschool children: Feasibility, reliability and practical recommendations for the PREFIT battery. J Sci Med Sport. 2016;19:910–5. 10.1016/j.jsams.2016.02.003. 26947061

[17] Cooper AR, Goodman A, Page AS, Sherar LB, Esliger DW, van Sluijs EMF, et al. Objectively measured physical activity and sedentary time in youth: the International children’s accelerometry database (ICAD). Int J Behav Nutr Phys Act. 2015;12:113. 10.1186/s12966-015-0274-5. PMC4574095 26377803

[18] Bull FC, Al-Ansari SS, Biddle S, Borodulin K, Buman MP, Cardon G, et al. World Health Organization 2020 guidelines on physical activity and sedentary behaviour. Br J Sports Med. 2020;54:1451–62. 10.1136/bjsports-2020-102955. PMC7719906 33239350

[19] Varni JW, Burwinkle TM, Seid M, Skarr D. The PedsQLy* 4.0 as a Pediatric Population Health Measure: Feasibility, Reliability, and Validity. Ambul Pediatr. 2003;3:329–41. 10.1367/1539-4409(2003)003<0329:tpaapp>2.0.co;2 14616041

[20] Dyer SL, Collins CT, Baghurst P, Saxon B, Meachan B. Insuflon Versus Subcutaneous Injection for Cytokine Administration in Children and Adolescents: A Randomized Crossover Study. J Pediatr Oncol Nurs. 2004;21:79–86. 10.1177/1043454203262680. 15125551

[21] Burdick P, Cooper S, Horner B, Cobry E, McFann K, Chase HP. Use of a subcutaneous injection port to improve glycemic control in children with type 1 diabetes. Pediatr Diabetes. 2009;10:116–9. 10.1111/j.1399-5448.2008.00449.x. 19175512

[22] Bohnert KL, Ditzenberger G, Bittel AJ, de las Fuentes L, Corti M, Pacak CA, et al. Resistance exercise training with protein supplementation improves skeletal muscle strength and improves quality of life in late adolescents and young adults with Barth syndrome: A pilot study. JIMD Rep. 2021;62:74–84. 10.1002/jmd2.12244. PMC8574175 34765401

[23] Bittel AJ, Bohnert KL, Reeds DN, Peterson LR, de las Fuentes L, Corti M, et al. Reduced Muscle Strength in Barth Syndrome May Be Improved by Resistance Exercise Training: A Pilot Study. JIMD Rep. 2018;41:63–72. 10.1007/8904_2018_102. PMC6122057 29654548

[24] Cade WT, Reeds DN, Peterson LR, Bohnert KL, Tinius RA, Benni PB, et al. Endurance Exercise Training in Young Adults with Barth Syndrome: A Pilot Study. JIMD Rep. 2017;32:15–24. 10.1007/8904_2016_553. PMC5362555 27295193

[25] Amedro P, Gavotto A, Huguet H, Souilla L, Huby A-C, Matecki S, et al. Early hybrid cardiac rehabilitation in congenital heart disease: the QUALIREHAB trial. Eur Heart J. 2024;45:1458–73. 10.1093/eurheartj/ehae085. PMC11032713 38430485

